# Evaluating the effect of digital game‐based nutrition education on anemia indicators in adolescent girls: A randomized clinical trial

**DOI:** 10.1002/fsn3.3120

**Published:** 2022-11-08

**Authors:** Omid Sabet Ghadam, Zahra Sohrabi, Manoosh Mehrabi, Mohammad Fararouei, Mansour Shahraki, Najme Hejazi, Cain C. T. Clark, Sanaz Mehrabani, Shirin Gerami, Mehran Nouri

**Affiliations:** ^1^ Department of Community Nutrition, School of Nutrition and Food Sciences Shiraz University of Medical Sciences Shiraz Iran; ^2^ Nutrition Research Center, School of Nutrition and Food Sciences Shiraz University of Medical Sciences Shiraz Iran; ^3^ Department of E‐learning in Medical Sciences, Virtual School, Center of Excellence for E‐leraning for Medical Sciences Shiraz University of Medical Sciences Shiraz Iran; ^4^ Department of Epidemiology, School of Health Shiraz University of Medical Sciences Shiraz Iran; ^5^ Department of Nutrition and Food Technology, Medical Faculty Zahedan University of Medical Sciences Zahedan Iran; ^6^ Centre for Intelligent Healthcare Coventry University Coventry UK; ^7^ Department of clinical Nutrition, School of Nutrition and Food Science Food Security Research Center, Isfahan University of Medical Sciences Isfahan Iran; ^8^ Students' Research Committee Shiraz University of Medical Sciences Shiraz Iran; ^9^ Health Policy Research Center, Institute of Health Shiraz University of Medical Sciences Shiraz Iran

**Keywords:** adolescents, digital games, iron deficiency anemia, nutrition education

## Abstract

Iron deficiency anemia is the most common type of micronutrient deficiency in the world. Adolescence represents a period of increased risk of iron deficiency. Therefore, we aimed to determine the impact of nutrition education by a digital game on markers of iron‐deficient anemia in adolescent girls. In this study, 176 adolescent girls were randomly dichotomized into the intervention and control groups. At the beginning and the end of the intervention, knowledge, attitude, and practice of both groups were assessed by a questionnaire. Girls in the intervention group received the necessary education through a digital game during a 14‐week period, while those in the control group received basic nutritional education through PowerPoint and pamphlets. Additionally, serum transferrin, serum iron, transferrin saturation, ferritin, CBC difference, and total iron binding capacity (TIBC) tests were checked. In this study, nutrition education significantly increased the level of knowledge, attitude, and practice of adolescent girls regarding their diet (*p* ˂ .05). Hemoglobin level was also significantly raised (*p* ˂ .05). However, no significant effect was observed on other markers of iron‐deficient anemia, such as serum iron, TIBC, and hematocrit, in the intervention group compared with the control group (*p* ˃ .05). The results of this study indicated the positive impact of nutrition education based on digital game on knowledge, attitude, and practice scores, as well as a significant difference in hemoglobin. It is recommended that educational games be designed for students in the future to promote health and nutrition information.

## Graphical abstract

We aimed to determine the impact of nutrition education by a digital game on markers of iron deficiency anemia in adolescent girls

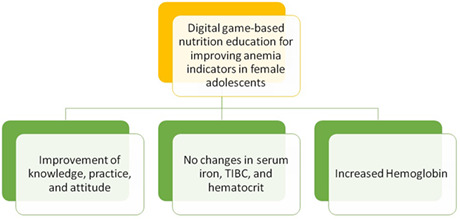

## 1 INTRODUCTION

Anemia is a condition that occurs when the volume of red blood cells (RBC) or the concentration of hemoglobin (Hb) in the blood decreases (Desalegn et al., 2014). Iron deficiency is considered as one of the most common causes of anemia, accounting for around half of all cases, where more than 2 billion individuals suffer worldwide (Stevens et al., 2013; Wu et al., 2016). As a nutritional deficiency, iron deficiency is a worldwide problem that mostly affects women of reproductive age, and those at the growth stage, such as infants and children (Unu, 2001).

According to the World Health Organization (WHO) report in 2001, the incidence of iron‐deficient anemia (IDA) among school‐aged children in developed countries was 5.9%, whereas it was 48.1% in developing countries (Unu, 2001). In the National Integrated Micronutrient Survey 2012, 9% of Iranian adolescents had anemia, of which 2.2% had iron deficiency anemia, where most were adolescent girls (Pouraram et al., 2018). Adolescent girls are 10 times more likely than boys to have iron deficiency anemia, possibly due to an increased need for growth spurts, nutritional deficiencies, weight‐loss diets, and menstrual bleeding (Derman et al., 2005). IDA is a serious public health issue in school‐aged children and associated with developmental delays, behavioral issues, and lower educational attainment, and therefore requires further attention, treatment, and prevention (Lozoff et al., 1991).

One of the most common methods of treatment and prevention of IDA is iron supplementation, which may increase the hematologic parameters of iron status, as well as nutritional education (Kapur et al., 2003). Studies have shown that nutritional education results are sustainable and can, in some cases, eliminate anemia. It is assumed that accurate guidance on topics such as food purchasing, food preparation and serving size, nutritional value of foods, and balanced diets provide sufficient information and motivation to make the right health choices (Kapur et al., 2003; Watson, 2007). One of the most active and contemporary learning strategies are digital games. While playing is a means of entertainment, it can also be instructive and constructive, where the learning materials are learned without pressure and with desire (Watson, 2007). Educational games can eliminate learning barriers to a large extent and provide better learning conditions for people (Watson, 2007). Indeed, research shows that educational games are effective on students' learning of concepts and have good stability (Kato, 2010), thus, it can be considered as an effective delivery method of nutrition education.

Considering the importance of iron deficiency and anemia in adolescent girls, the lack of studies in this field, the need for planning to implement further interventions due to the serious complications of iron deficiency anemia, and the importance of gamed‐based educations, this study aimed to improve the nutritional behavior related to anemia and iron deficiency using digital games in high school adolescent girls in Saravan, Iran (an Iranian city in Sistan and Baluchestan province with lower socioeconomic status and high rates of nutritional deficiencies, especially in the adolescent girls). According to a previous study conducted in the same population, the prevalence of anemia and iron deficiency anemia was 24% and 12.6%, respectively (Ghadam et al., 2020).

## 2 METHODS

### 2.1 Participants

This randomized clinical trial was conducted in the period of October 2018 to March 2020, on 176 randomly selected adolescent girl students aged 10–19 years in Saravan city. In this study, Hb <12 g/dl was considered an index of anemia, and ferritin <12 μg/dl was considered an index of iron deficiency anemia. Because the prevalence of iron deficiency anemia in this region was not as high as the required sample size for our intervention, 176 adolescent girls with the lowest Hb levels were selected and then randomly allocated into the intervention and control groups, respectively. The inclusion criteria for the recruitment of students for this study were: (1) school‐age girls aged between 10 and 19 years old; (2) those who can use, and have access to, a smartphone or tablet; (3) no apparent skeletal, chronic, or infectious diseases; (4) having regular menarche for at least 1 year; (5) no heavy bleeding during menstruation; (6) no history of iron or other vitamin or mineral supplementation before and during the study. The exclusion criteria were: (1) becoming ill during the study that required special treatment; (2) students who were transferred to other schools were also excluded from the study. The study was approved by the Ethics Committee of Shiraz University of Medical Sciences (SUMS). The study conformed to the Declaration of Helsinki and the Good Clinical Practice Guidelines of International Council for Harmonization of Technical Requirements for Pharmaceuticals for Human Use. Also, the design protocol was registered in the Iranian Registry of Clinical Trials under the number IRCT20190610043857N1 so that all people can have access to it.

### 2.2 Study design

In a previous study on the same population, using cluster sampling method, 45 schools were randomly selected and 3 classes were chosen from each school, and then, 463 students were randomly selected to investigate the prevalence of iron deficiency anemia in the study population (Ghadam et al., 2020). This study was performed as a randomized clinical trial for 14 weeks on a screened sample of the students (those with the lowest levels of Hb in the main sample). We used the standard formula for parallel design randomized controlled trials to calculate the required sample size by considering a study power of 80% and type I error of 5% (α = 0.05) and type II error of 80% (β = 0.8) and 10% dropout rate to calculate sample size. In total, 176 adolescent girls with the lowest levels of Hb were randomly divided into two groups including 88 girls in the intervention group and 88 in the control group. The girls in the intervention group received the predefined training through digital games during the 14 weeks of the study. The control group also received the same information through PowerPoint presentation and pamphlets. The game consisted of seven stages and each stage was opened for students every other week, such that the whole game was finished in 14 weeks. During the game, information about the high‐risk groups, causes, symptoms of iron deficiency anemia, the amount of iron required in different groups required to prevent iron deficiency anemia, and iron‐rich food sources were provided to the gamers. In each stage, the gamer was allowed to play for a maximum of 45 min, and after 45 min, a participant was not able to play for at least 3 h.

At the beginning and end of the intervention for both groups, the level of knowledge, attitude, and practice were assessed by a specially designed questionnaire. Knowledge was assessed by 21 questions, attitude was assessed by 19 questions based on the Likert scale, and performance status was assessed based on the average score obtained in response to 11 questions (Falahi et al., 2010). The content of the questions was mostly related to the definition of iron deficiency anemia, high‐risk groups, contributors, symptoms, side effects, and sources of iron‐containing foods. The reliability and content validity of the questionnaire were measured using test–retest approach and expert's opinion (two nutritionists). Also, a 3‐day food recall was completed, and anthropometric measures were obtained from all participants. The weight was measured in minimal clothing and without shoes. Height was measured with nonstretchable tape in standing position. Using these measurements, body mass index (BMI) was calculated by dividing body weight in kilograms by height in meters squared (kg/m^2^). Waist circumference (WC) was assessed with an inelastic tape on the approximate midpoint between the lower margin of the last palpable rib and the top of iliac crest.

At the end of 14th week, anthropometric and biochemical measurements such as Hb, ferritin, transferrin, serum iron, and total iron‐binding capacity (TIBC) were rechecked in both groups.

### 2.3 Assessment of blood samples

Five milliliters of venous blood were collected from participants and the blood sample was put in two separate containers to measure Hb (1 cc of blood in tubes containing EDTA anticoagulant) and the rest was poured into the hemolysis tube to separate the serum and it was stored in the −75°C freezer before final measurements. Hemoglobin and ferritin were measured to evaluate iron deficiency anemia. Hemoglobin level <12 g/dl was considered as anemia, and if ferritin level was <12 g/dl, iron deficiency anemia was diagnosed (Zare, 2002). To evaluate the effect of the intervention after 14 weeks, laboratory tests of transferrin, serum iron, transferrin saturation, ferritin, CBC difference, and TIBC were used. For biochemical tests of transferrin, serum iron, and TIBC, Pars Azmoon Kit (Iran) and calorimetric method (auto analyzer) were used; for ferritin, Pishtaz Company kit (Iran) and ELISA method were used; and for CBC difference test, cell counter was used. Transferrin saturation was calculated from the distribution of serum iron to TIBC. TruCal U test was also performed using Pars Azmoon Kit for standardization.

### 2.4 Statistical analysis

The final data were analyzed using SPSS software version 22, Inc. The distributions of the study variables in the participants were compared using the Kolmogorov–Smirnov test. To compare in the quantitative study variables before and after the intervention, a paired *t* test was used in each group. To compare the changes in the two groups, an independent samples *t* test was applied. For skewed data, Wilcoxon signed rank test and Mann–Whitney *U* tests were applied for inter‐ and intragroup comparisons, respectively. The chi‐square test was applied to evaluate the relationship between iron deficiency anemia and qualitative variables. The *p*s < .05 were considered significant.

## 3 RESULTS

**FIGURE 1:**
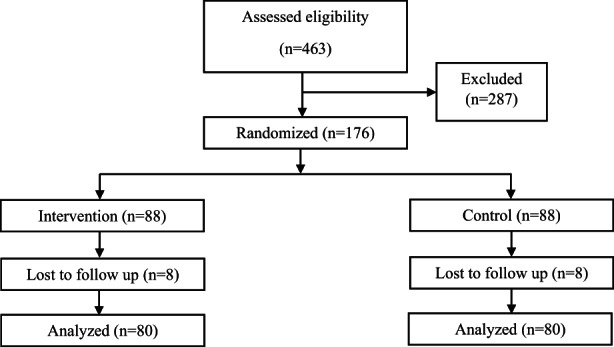
Flowchart of participants recruitment

Among the 176 adolescent girls in the study, 8 participants declined to continue their collaboration in each group, and were excluded. Finally, 160 participants completed the study (Figure 1). The participants' compliance was high, and there was no statistically significant difference between the study groups.

The baseline characteristics and the measured parameters of the participants are shown in Table 1. In terms of baseline anthropometric parameters, markers of iron deficiency anemia, knowledge, attitude, and practice, there were no significant differences between both groups, except in transferrin level (*p* < .001).

**TABLE 1:** Baseline characteristics and the measured parameters of the study participants

Characteristics^a^	Intervention (*n* = 80)	Placebo (*n* = 80)	*p*
Weight (kg)	47.97 ± 11.79	46.36 ± 9.27	.49
Height (cm)	151.10 ± 10.85	150.12 ± 8.51	.25
BMI (kg/m^2^)	21.03 ± 4.95	20.59 ± 4	.74
Waist (cm)	69.62 ± 11.58	67.52 ± 7.24	.10
Hb (g/dl)	11.84 ± 1.21	11.96 ± 0.93	.55
Ferritin (ng/ml)	31.74 ± 27.01	23.18 ± 15.58	.12
Transferrin (mg/dl)	358.98 ± 75.32	315.11 ± 50.47	**<.001**
Iron (μg/dl)	70.95 ± 25.20	65.31 ± 18.25	.25
TIBC (μg/dl)	251.78 ± 7.21	256 ± 30.33	.22
Transferrin saturation (%)	28.78 ± 7.21	28.78 ± 7.21	.06
Hematocrit (%)	34.63 ± 3.10	34.90 ± 2.60	.81
MCV (fL)	76.85 ± 10.65	75.05 ± 9.54	.10
MCH (pg)	26.25 ± 4.4	26.61 ± 4.31	.22
MCHC (g/dl)	34.38 ± 2.20	34.58 ± 1.79	.77
Knowledge score	46.01 ± 4.09	45.58 ± 5.43	.35
Attitude score	60.70 ± 6.92	57.69 ± 12.94	.28
Practice score	26.56 ± 5.08	27.85 ± 7.25	.54

Abbreviations: BMI, body mass index; Hb, hemoglobin; MCH, mean corpuscular hemoglobin; MCHC, mean corpuscular hemoglobin concentration; MCV, mean corpuscular volume; TIBC, total iron‐binding capacity.

Bold indicates significant *p*‐values.

^a^ Nonparametric analysis was used for variables and data are expressed as mean ± SD. *p* < .05 was considered significant.

The pre and post nutrient intakes are presented in the Table 2. Regarding nutrient intake at the baseline of the study, there was no statistically significant difference between both the groups, except for fat (*p* = .001), cholesterol (*p* ˂ .001), potassium (*p* = .009), and vitamin E (*p* = .004) intakes. At the end of the study, we observed a significant difference among two groups for energy (*p* ˂ .001), fiber (*p* ˂ .001), phosphorus (*p* = .01), iron (*p* = .006), potassium (*p* = .04), and vitamin A (*p* = .004) consumption. Also, intergroup analysis showed a significant difference in energy (*p* = .04), fiber (*p* = .004), and iron (*p* ˂ .001) intake in the intervention group, and energy and fat in the control group (*p* = .02 for both) at the end of the study compared to the baseline.

**TABLE 2:** Baseline and after nutrient intakes of the study participants

Characteristics^a^	Intervention (*n* = 80)	Placebo (*n* = 80)	Intragroup *p* value
Energy (Kcal)			
Before	2135.85 ± 244.44	2168.91 ± 256.16	.33
After	2196.08 ± 224.29	2043.35 ± 299.73	**˂.001**
Intragroup *p*	**.04**	**.02**	
Protein (g)			
Before	61.05 ± 17.25	63.63 ± 10.67	.16
After	64.63 ± 16.48	62.22 ± 10.91	.14
Intragroup *p*	.20	.07	
Carbohydrate (g)			
Before	273.34 ± 48.97	262.80 ± 48.12	.31
After	266.15 ± 48.84	267.59 ± 54.48	.57
Intragroup *p*	.38	.51	
Fat (g)			
Before	92.06 ± 19.10	101.22 ± 17.69	**.001**
After	93.75 ± 18.64	92.71 ± 23.34	.37
Intragroup *p*	.48	**.02**	
Fiber (g)			
Before	13.16 ± 7.94	11.08 ± 4.63	.27
After	17.11 ± 10.98	11.58 ± 3.04	**˂.001**
Intragroup *p*	**.004**	.15	
Cholesterol (mg)			
Before	141.07 ± 64.59	183.79 ± 55.06	**<.001**
After	143.75 ± 63.35	181.49 ± 52.57	.80
Intragroup *p*	.49	.40	
Calcium (mg)			
Before	922.08 ± 341.14	1011.08 ± 293.95	.38
After	1014.49 ± 360.95	952.98 ± 243.93	.10
Intragroup *p*	.17	.43	
Phosphorus (mg)			
Before	989.83 ± 313.81	1019.17 ± 170.60	.88
After	1051.24 ± 308.61	963.97 ± 180.35	**.01**
Intragroup *p*	.19	.10	
Iron (mg)			
Before	15.94 ± 9.55	18.12 ± 12.15	.41
After	22.71 ± 13.70	17.71 ± 11.52	**.006**
Intragroup *p*	**˂.001**	.79	
Potassium (mg)			
Before	2394.69 ± 598.41	2644.95 ± 653.11	**.009**
After	2623.01 ± 725.04	2477.82 ± 564.08	**.04**
Intragroup *p*	.35	.06	
Vitamin A (RE)			
Before	10.34 ± 6.32	9.28 ± 2.79	.22
After	10.58 ± 6.27	8.50 ± 3.33	**.004**
Intragroup *p*	.55	.12	
Vitamin E (mg)			
Before	30.43 ± 9.66	34.08 ± 10.51	**.004**
After	30.84 ± 9.10	32.57 ± 13.44	.83
Intragroup *p*	.84	.49	
Vitamin C (mg)			
Before	65.57 ± 54.55	87.75 ± 78.69	.13
After	86.36 ± 71.79	73.73 ± 68.91	.14
Intragroup *p*	.84	.06	
Vitamin D (μg)			
Before	0.47 ± 0.45	0.52 ± 0.68	.23
After	0.50 ± 0.48	0.51 ± 0.67	.54
Intragroup *p*	.46	.75	

Bold indicates significant *p*‐values.

^a^ Nonparametric analysis was used for variables and data are expressed as mean ± SD. *p* < .05 were considered significant.

The primary and secondary outcomes changes are shown in Table 3. Weight was significantly reduced in the intervention group (*p* = .003), however, there was a significant increase in their height during the study (*p* = .01). Also, a significant increase was observed in weight (*p* = .008) and WC (*p* = .02) in the intervention group. However, anthropometric changes between groups were not significant (*p* ˃ .05). Change in Hb was significantly different between groups (*p* = .03). Furthermore, intergroup analysis showed that there was a significant increase in Hb and ferritin in both groups (*p* ˂ .001), but changes were higher in the intervention group compared to the control group; however, we observed significant increase in iron (*p* = .004), transferrin (*p* = .02), MCV (*p* = .03), MCH (*p* = .01), and MCHC (*p* = .01) only in the control group.

**TABLE 3:** Effects of the nutrition education on the measured variables in the study groups

Characteristics^a^	Intervention group (*n* = 80)			Control group (*n* = 80)			Intervention effect
	After	Change^b^	Intragroup *p* *	After	Change	Intragroup *p* *	*p* ^c^
Weight (kg)	48.01 ± 11.77	−0.04	**.003**	46.54 ± 9.11	0.18	**.008**	.38
Height (cm)	151.21 ± 10.90	0.11	**.01**	150.21 ± 8.48	0.09	.05	.68
BMI (kg/m^2^)	21.05 ± 4.91	−0.02	.71	20.60 ± 3.95	0.01	.88	.88
Waist (cm)	69.71 ± 5.01	0.09	.05	67.67 ± 7.12	0.15	**.02**	.58
Hb (g/dl)	12.26 ± 0.87	0.42	**<.001**	12.18 ± 0.74	0.22	**<.001**	**.03**
Ferritin (ng/ml)	33.96 ± 26.23	2.22	**<.001**	24.89 ± 14.89	1.71	**<.001**	.16
Transferrin (mg/dl)	351.87 ± 98.85	−7.11	.22	314.51 ± 68.22	−0.6	.37	.15
Iron (μg/dl)	74.75 ± 25.98	3.83	.35	65.91 ± 16.01	0.6	**.004**	.17
TIBC iron (μg/dl)	248.96 ± 48.97	2.21	.73	254.47 ± 35.39	−1.53	.38	.74
Transferrin saturation (%)	26.64 ± 6.49	0.86	.29	28.99 ± 6.11	0.18	**.02**	.71
Hematocrit (%)	35.36 ± 2.32	0.73	.56	34.90 ± 1.78	0.01	.72	.27
MCV (fL)	77.50 ± 7.66	0.92	.30	76.48 ± 7.39	1.43	**.03**	.50
MCH (pg)	27.25 ± 3.48	0.64	.11	26.99 ± 3.62	0.74	**.01**	.77
MCHC (g/dl)	34.73 ± 2.15	0.35	.21	35.13 ± 1.69	0.55	**.01**	.47
Knowledge score	56.16 ± 3.98	10.15	**<.001**	50.57 ± 6.95	5.2	**<.001**	**<.001**
Attitude score	71.05 ± 6.04	10.35	**<.001**	61.01 ± 9.67	3.31	**.002**	**<.001**
Practice score	34.65 ± 4.05	8.09	**<.001**	31.06 ± 4.81	3.21	**<.001**	**<.001**

Abbreviations: BMI, body mass index; Hb, hemoglobin; MCH, mean corpuscular hemoglobin; MCHC, mean corpuscular hemoglobin concentration; MCV, mean corpuscular volume; TIBC, total iron‐binding capacity.

Bold indicates significant *p*‐values.

* *p* values denote the significance of within‐group changes with Wilcoxon test.

^**a**^ Nonparametric analysis was used for variables.

^b^ Data are expressed as posttreatment value less than the pretreatment value and are given as mean.

^c^ *p* values denote the significance of between‐group changes (the intervention group compared with the control) with Mann–Whitney test. *p* < .05 were considered significant.

Significant differences were shown between groups regarding change in the scores related to knowledge (*p* ˂ .001), attitude (*p* ˂ .001), and practice (*p* ˂ .001). Also, knowledge (*p* ˂ .001 for both groups), attitude (*p* ˂ .001 in the intervention and *p* = .002 in the control group) and practice score (*p* ˂ .001 for both groups) increased significantly in both groups, however, these changes were higher in the intervention group.

## 4 DISCUSSION

Results of this study showed that nutrition education using digital games elicits a significant improvement in the scores of knowledge, attitude, practice, and Hb level of high school girls with obesity.

Educational programs during adolescence play an important role in increasing knowledge, improving attitudes, and practice. This study revealed that implementing a nutritional education would effectively improve knowledge, attitudes, and practice regarding iron deficiency anemia among school‐age girls in both groups. The mentioned factors were significantly improved in the digital game‐based nutrition education group compared with control group which could show the greater impact of educational games than the traditional methods on level of knowledge, attitude, and practice of students. According to our findings, in digit game arm, energy intake significantly increased postintervention. Also, this change was significant compared with control group. However, protein intake increased, and carbohydrate intake decreased, after the intervention in digit game group, but the changes were not significant. So, the energy supply from protein was increased after intervention in digit game arm, and using body analysis, the change in fat‐free mass and fat mass was more evident than using the relatively obtuse measures of BMI and weight. Clearly, change in physical activity level should be considered in weight management, for instance, energy intake decreased postintervention in the control group, but weight increased after intervention group, so it is possible that this variation was due to the physical activity changes. Accordingly, teaching adolescents to increase their physical activity can help weight management. In accordance with the result of this study, a recent trial showed that nutritional education significantly improved practice, attitude, and knowledge about iron deficiency among adolescent girls (Abu‐Baker et al., 2021). Another recent study demonstrated that nutrition education, using the PRECEDE model, yielded improvements in attitude, knowledge, preventive behaviors, and self‐efficacy, compared with control group, on iron deficiency anemia among female students during a 4‐month intervention (Khani Jeihooni et al., 2021). Thus, it appears that digital games can initiate behavioral changes and increase motivation and knowledge in people, especially in adolescents and young people who more easily accept new technologies (Khani Jeihooni et al., 2021).

The results of anthropometric evaluations showed that the mean weight, BMI, and waist circumference, before and after the educational intervention, were not statistically significant between the intervention and control groups. However, weight change reduced in the digital‐based nutrition education group after intervention, but the changes were not significant between groups. Lack of changes in the anthropometric indices may be attributable to the fact that, in this study, the education was not provided with the aim of weight management. In concordance with the results of this study, a recent trail in Iran showed that nutrition education intervention among school‐aged children, using social cognitive theory (SCT) for 7 months, had no significant effect on BMI and weight circumference compared with the control group (Bagherniya et al., 2017). Also, another study demonstrated that a 12‐month multicomponent intervention, including nutrition education and other tailored school‐based interventions, did not change BMI compared with control group (Lubans et al., 2012). Additionally, a meta‐analysis study in 2014 revealed that school‐based nutrition education and physical activity had no significant effect on BMI in adolescent and children (Guerra et al., 2014). However, some studies showed that nutrition education intervention reduced anthropometric indices (Jorvand et al., 2016; Normayanti & Prayitno, 2020). This study was designed to educate participants for changing the intake of iron‐rich foods and improve dietary patterns in prevention of anemia. Indeed, in this study, no training was conducted to elicit change the energy intake in order to change weight and other anthropometric indices, so it seems that is reasonable and expected not to find any difference between the intervention and control groups in terms of anthropometric indices.

According to the results of this study, nutrition education based on digital games resulted in increases in some nutrients intake compared to the control group, including protein, fat, phosphorus, iron, potassium, and vitamin C. In the educational intervention program based on digital game methods, in this study, all food sources containing iron or iron absorption enhancers and absorption inhibitors were trained/taught. Hence, consumption of iron‐containing foods, including animal proteins that are high in protein, fat, iron, and phosphorus, were enhanced following nutrition education, and the changes in the dietary intake of these nutrients seems logical compared with the control group. Moreover, regarding iron absorption enhancement, dietary sources of vitamin C such as fruits and vegetables that are rich sources of potassium and vitamin C were encouraged and a higher intake of potassium and vitamin C happened after the digit game intervention versus control arm; although this change did not reach statistical significance for vitamin C intake. On the other hand, a significant difference between two groups indicates the positive effect of digital games on increasing nutritional knowledge and use of this knowledge. Digital games enhance the learning process and create motivation and attraction in people, so these games can be useful tools to promote healthy eating related behaviors in people.

The results of this study about the effect of nutrition education on markers of iron deficiency anemia showed that nutrition education caused a significant increase in Hb and ferritin levels in both the intervention and control groups, which was more pronounced in the intervention group compared with control group; although nutrition education did not have a significant effect on other markers of iron deficiency anemia such as ferritin, serum iron, TIBC, and hematocrit. Improvement in some markers of iron deficiency anemia was due to improvement in nutrients intake after the digit game intervention. According to our results, iron intake increased significantly in the digit game group compared with placebo arm, postintervention. In addition, increase in vitamin C intake, that is an iron absorption enhancer, was detected in digit game arm, though this change was not statistically significant versus the control group. A recent trial showed that nutrition education, using the PRECEDE model for 4 months, could lead to significantly increased ferritin concentration in the intervention group compared with the control group. Also, Hb levels increased after 4‐month intervention compared with the control group; albeit only marginally significant (Khani Jeihooni et al., 2021). Another study showed that ferritin concentration increased significantly after nutrition education compared with baseline value among 6–14 years old students (García‐Casal et al., 2011). The results of a study by Alaofé and colleagues demonstrated the effective role of nutrition education on ferritin, Hb, and iron concentration, while it was not effective on TIBC and transferrin levels (Alaofé et al., 2009). Furthermore, another study revealed that though nutrition education for 2 months improved nutritional knowledge score among adolescent girls, it had no effect on markers of anemia, including ferritin and Hb (Amani & Soflaei, 2006). The result of another study showed that nutrition education increased ferritin level among iron‐depleted children and increased the intake of fruit juice, which was a rich dietary vitamin C source, which could possibly increase ferritin level (Khoshnevisan et al., 2004). In this study, dietary intake of nutrients such as iron and vitamin C increased in the intervention group compared with the control group, and that it may be effective on improving marker of anemia such as ferritin and Hb. Also, within‐group comparisons showed that the mean of MCV, MCH, and MCHC, in both intervention and control groups, increased. Moreover, this increase was significant in the control group, so it should be noted that traditional education appeared to be effective in this group, while the increase of these markers may be influenced by other dietary factors.

However, the lack of changes in anemia indicators could be justified according to the short duration of the study. Nutrition education is the most basic and important strategy to achieve changes in people's eating habits, and it can affect people's knowledge, attitude, and practice, but its effect on biochemical markers requires long‐term interventions. Nutrition education using digital games has been effective in improving anemia, however, some markers of iron deficiency anemia in the intervention group, and some in the control group, changed in this study. Therefore, achieving a firm conclusion regarding the difference in the effect of the two methods of education is not straightforward, and requires further investigations.

One of the strengths of this study was the novel use of digital games for nutrition education. The findings of studies showed that digital educational games can enhance the learning process, motivate, and increase self‐confidence and concentration. However, one of the limitations of this study was the limited age range of participants, and some results, such as fatigue, anxiety, and mental state, were inconclusive. Nevertheless, this was beyond the control of the research.

## 5 CONCLUSION

Nutrition education with a digital game could positively affect the knowledge, attitude, and practice of the adolescent girls regarding iron deficiency anemia and positively impact dietary intake of protein, fat, iron, vitamin C, and potassium. Nutrition education, over a 12‐week period, may enhance Hb levels without affecting other indicators of IDA in adolescent girls. Furthermore, it is suggested that this study may be performed on different ages and genders who are exposed to iron deficiency for a longer period of time. Considering the potential benefits of digital games in education, it is suggested that educational games be created, with the cooperation of education specialists, physicians, nutritionists, and game designers, to promote health and nutrition information in students and children, especially in less‐developed countries.

## Data Availability

The data supporting the finding of this study are available from the corresponding author through reasonable request.
